# Time-course transcriptome analysis of human cellular reprogramming from multiple cell types reveals the drastic change occurs between the mid phase and the late phase

**DOI:** 10.1186/s12864-017-4389-8

**Published:** 2018-01-03

**Authors:** Akihiro Kuno, Ken Nishimura, Satoru Takahashi

**Affiliations:** 1 Department of Anatomy and Embryology, Faculty of Medicine, University of Tsukuba, 1-1-1 Tennodai, Tsukuba, Ibaraki 305-8575 Japan; 2 Laboratory of Gene Regulation, Faculty of Medicine, University of Tsukuba, 1-1-1 Tennodai, Tsukuba, Ibaraki 305-8575 Japan; 3 Ph.D Program in Human Biology, School of Integrative and Global Majors, University of Tsukuba, 1-1-1 Tennodai, Tsukuba, Ibaraki 305-8575 Japan

**Keywords:** Induced pluripotent stem cell, Cellular reprogramming, Time-course gene expression, Transcriptional factor, Transcriptional factor regulatory network

## Abstract

**Background:**

Human induced pluripotent stem cells (hiPSCs) have been attempted for clinical application with diverse iPSCs sources derived from various cell types. This proposes that there would be a shared reprogramming route regardless of different starting cell types. However, the insights of reprogramming process are mostly restricted to only fibroblasts of both human and mouse. To understand molecular mechanisms of cellular reprogramming, the investigation of the conserved reprogramming routes from various cell types is needed. Particularly, the maturation, belonging to the mid phase of reprogramming, was reported as the main roadblock of reprogramming from human dermal fibroblasts to hiPSCs. Therefore, we investigated first whether the shared reprogramming routes exists across various human cell types and second whether the maturation is also a major blockage of reprogramming in various cell types.

**Results:**

We selected 3615 genes with dynamic expressions during reprogramming from five human starting cell types by using time-course microarray dataset. Then, we analyzed transcriptomic variances, which were clustered into 3 distinct transcriptomic phases (early, mid and late phase); and greatest difference lied in the late phase. Moreover, functional annotation of gene clusters classified by gene expression patterns showed the mesenchymal-epithelial transition from day 0 to 3, transient upregulation of epidermis related genes from day 7 to 15, and upregulation of pluripotent genes from day 20, which were partially similar to the reprogramming process of mouse embryonic fibroblasts. We lastly illustrated variations of transcription factor activity at each time point of the reprogramming process and a major differential transition of transcriptome in between day 15 to 20 regardless of cell types. Therefore, the results implied that the maturation would be a major roadblock across multiple cell types in the human reprogramming process.

**Conclusions:**

Human cellular reprogramming process could be traced into three different phases across various cell types. As the late phase exhibited the greatest dissimilarity, the maturation step could be suggested as the common major roadblock during human cellular reprogramming. To understand further molecular mechanisms of the maturation would enhance reprogramming efficiency by overcoming the roadblock during hiPSCs generation.

**Electronic supplementary material:**

The online version of this article (10.1186/s12864-017-4389-8) contains supplementary material, which is available to authorized users.

## Background

Human induced pluripotent stem cells (hiPSCs) have revolutionized not only stem cell research but also clinical medicine by advancing cell therapy, disease modeling, and drug discovery. However, the reprogramming process is still inefficient and establishment of high-quality hiPSCs is unreliable regardless of many developed reprogramming methods to increase efficiency and safety [1, 2]. Therefore, to elucidate underlying mechanisms of reprogramming procedure by unveiling its roadblock has important implication for the hiPSCs generation.

Previous studies conducted time-course gene expression analyses during reprogramming using mouse embryonic fibroblasts (MEFs) [3, 4]. These studies suggested the progression of reprogramming is broadly divided into three phases: initiation, maturation, and stabilization. Briefly, reprogramming is initiated with mesenchymal-to-epithelial transition (MET), one of the hallmark events of initiation. Next, the intermediate reprogramming cells obtain expressions of a subset of pluripotency genes by exogenous transgene-dependent manner for maturation. Finally, the reprogramming cells gain transgene-independent stem cell property through stable expression of pluripotent genes at stabilization [3–5]. Furthermore, a recent work illustrated reprogramming roadmaps of MEFs with higher resolution by using cell surface marker based subpopulation analysis. The results indicated that suppression of mesenchymal genes is followed by transient upregulation of epidermis related genes whose inactivation soon turns on pluripotency genes [6, 7].

However, the characteristics and the timing of hiPSCs reprogramming events have been reported to be different from mouse, although iPSCs can be generated by the induction of the same transcription factors [8]. For example, MET occurs later in human reprogramming process, which is when exogenous OSKM (OCT4, SOX2, KLF4, and c-MYC) becomes suppressed and endogenous OCT4 starts to appear [9]. In addition, the pluripotent states are referred differently for human and mouse iPSCs, ‘primed’ and ‘naïve’, respectively [10, 11]. Because the understanding of human cell reprogramming process is still limited compared to mice, to explore reprogramming process in human cells as comprehensively as in mouse cells is of the utmost importance.

Although the current insights of cellular reprogramming of hiPSCs were confined to fibroblasts, hiPSCs have been established from multiple somatic cell types including dermal fibroblasts [12, 13], adipose-derived stem cells [14–17], neural stem cells [18], hepatocytes [19], amniotic fluid-derived cells [20, 21], epithelial cells [22–24], melanocytes [25] and peripheral blood cells [26–28]. Notably, a recent research reported that all five OSKM-induced human somatic cell types exhibited transiently similar transcriptome profile which resembled a primitive streak [29]. These facts suggest that partially common pathway in hiPSCs reprogramming might exist across multiple cell types. Furthermore, a recent study indicated that the maturation, from day 7 to 15 upon OSKM transduction in human dermal fibroblasts (HDFs), is a major roadblock of reprogramming process [30]. Thus, we aimed to differentiate reprogramming process shared in various human cell types in order to evaluate whether maturation is a common roadblock in other cell types or not.

For the purpose, we extracted dynamically expressed genes in five different human somatic reprogramming cell types from time-course microarray dataset [29]. Next, we divided the genes into five clusters according to gene expression patterns and functionally characterized each cluster. Lastly, we inferred and snapshotted transcription factor (TF) activity during reprogramming process. The results obtained in this work suggested reprogramming was consistently driven through three phases, in all five-cell types including fibroblasts, adipose-derived stem cells, astrocytes, bronchial epithelial cells and prostate epithelial cells. Furthermore, the maturation can be proposed as the common roadblock of reprogramming in five cell types.

## Methods

### Microarray data

**Fig. 1:**
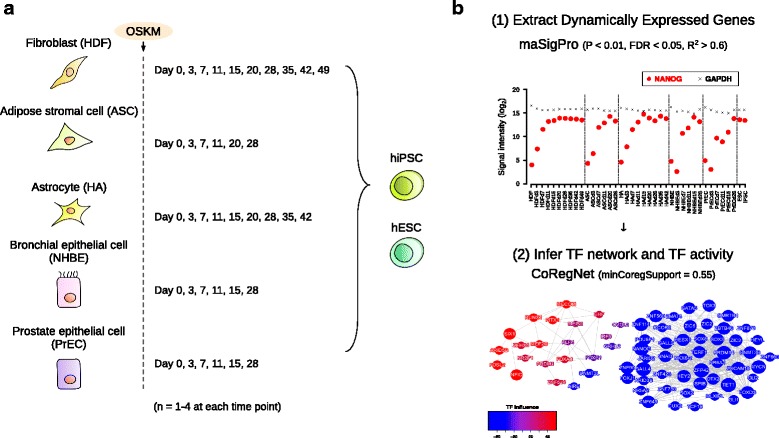
Schema of the experiment. **a** Samples of microarray dataset used in this study were obtained from GSE50206 [29], which includes five human somatic cells during reprogramming and two stem cells. **b** Experimental workflow. NANOG and GAPDH were typical examples of dynamically expressed genes and statically expressed genes in reprogramming, respectively. The filtration method extracted NANOG and eliminated GAPDH. The main parameters were indicated in parentheses (See Methods for the detailed description)

To find conserved genes with dynamic expression from various human cell types in cellular reprogramming, we used a dataset from Gene Expression Omnibus under the accession number GSE50206 [29]. It contains time-course microarray data of five human somatic cell types: HDF (fibroblast), ASC (adipose-derived stem cell), HA (astrocyte), NHBE (bronchial epithelial cell) and PrEC (prostate epithelial cell) during cellular reprogramming, and two stem cells: hiPSC, and hESC (Fig. 1a). All sample records (GSM) used in the study were listed in Additional file 1: Table S1.

### Data processing, gene selection and transcription factor activity inference

The raw signals from dataset were processed by log_2_ transformation and quantile normalization. We used the limma package for quantile normalization in R using Bioconductor [31]. The signal intensities of each gene in the biological replicates were averaged. Next, to extract dynamically expressed genes across all cell types during reprogramming process, we individually proceeded the maSigPro package [32] in each cell and screened genes which showed the significance in all five cell types (*P*-value <0.01, FDR < 0.05, R^2^ > 0.6). The filtration yielded 3615 extracted genes (Fig. 1b). When multiple probes were annotating the same extracted genes that are extracted, the signals were averaged.

After extracting 3615 genes in five cell types during reprogramming, we applied the CoRegNet package [33] to infer the activity of transcription factor (TF) in the reprogramming process. The CoRegNet infers cooperative TF network and scores TF influences with the h-LICORN algorithm by using TFs and target genes expression profiles (Fig. 1b) [34]. To reconstruct regulatory networks, we set the parameter of minCoregSupport as 0.55 due to the limitation on computational memory, where the parameter indicates how frequently the set of co-regulators appears in the dataset (Fig. 1b).

To visualize the influence of representative TFs, we extracted 71 TFs which have significant pairs of co-regulators (alpha <0.01) and were found many times in the net (more than one hundredth of the maximum number of gene regulatory network). These are default parameters in CoRegNet package.

### Principal component analysis (PCA) and Hierarchical Clustering Analysis (HCA)

**Fig. 2:**
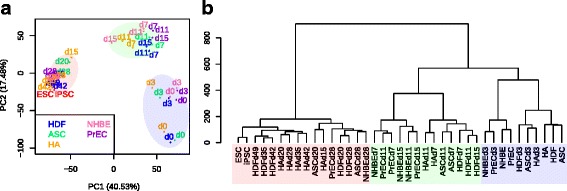
Transcriptome analyses of 3615 extracted genes from each reprogrammed-cell type showed three clusters by the degree of relatedness. **a** Principal component analysis. Each cell type was colored as followings: HDF (blue), ASC (green), HA (orange), NHBE (pink), PrEC (purple), hiPSCs (red), and hESCs (red). The numbers indicated that the days of the RNA collection after OSKM induction. **b** Hierarchical cluster analysis of each cell type. **a**, **b** The early phase, mid phase, and late phase were labeled as the translucent blue, green, and red colors, respectively

**Fig. 3:**
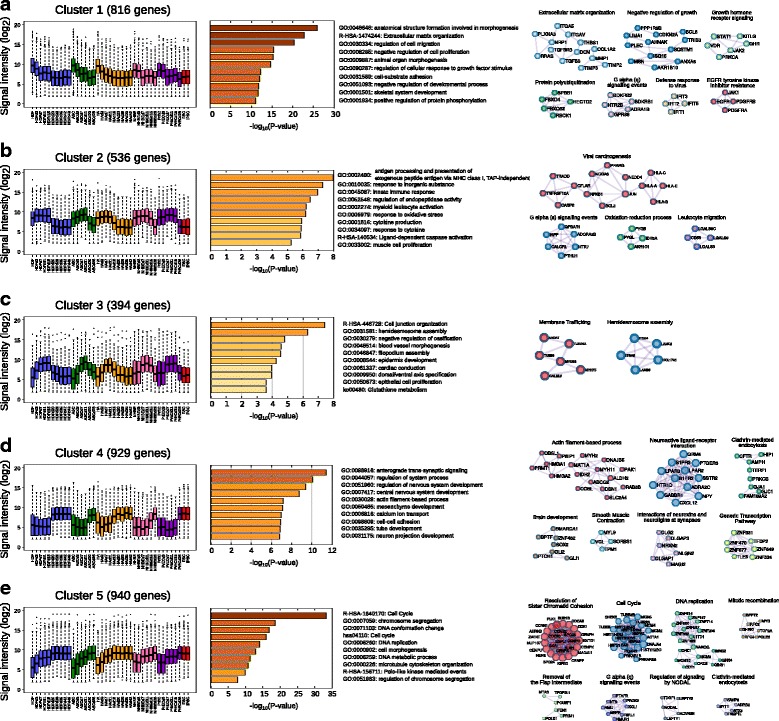
Common gene expression dynamics and their functional annotations. The left panels showed gene expression patterns. The middle panel described functional gene enrichment analysis. The annotations of GO, R-HSA, and both ko and hsa indicated their identifiers from Gene Ontology, Reactome, and KEGG, respectively. Right panels illustrated protein-protein interaction (PPI) network. **a** Cluster 1 contains 816 genes which have high expression in the early phase. **b** Cluster 2 contains 536 genes which have high expression in the early and mid phase. **c** Cluster 3 contains 394 genes with transient upregulation in the mid phase. **d** Cluster 4 contains 929 genes with sharp upregulation in the late phase. **e** Cluster 5 contains 940 genes with the gradual expression increase

In Fig. 2, we used correlation matrix to find the components in PCA. HCA was performed using Euclidean distance and Ward’s linkage method. In Fig. 3, HCA was performed using cosine similarity and Ward’s linkage method.

### Pathway, Gene Ontology (GO), and Protein-protein Interaction (PPI) enrichment analysis

For functional annotation of gene sets, we used Metascape (http://metascape.org) to find top 10 clusters with the representative enriched terms of Reactome and GO Biological Processes [35]. Connected PPI network was inferred by MCODE algorithms [36] with Metascape default parameters. We selected significant enriched MCODE clusters which consist of more than four nodes.

### Data analysis of histone modification

In Additional file 2: Figure S3, we analyzed the ChIP-seq data which contains H3K79me2 and H3K27me3 of fibroblasts differentiated from H1 ESCs (dH1f), at day 6 of OSKM retroviruses infected fibroblasts derived from dHIf and H1 ESCs (GSE35791) [37]. ChIP-Seq signal was quantified as total number of reads per million in the region of interest. We extracted genomic regions which have top 0.1% signal intensity and annotated the nearest genes within a range of 10 kb from the TSS.

## Results

### Three distinct transcriptomic states exist during cellular reprogramming in various cell types

To analyze the relatedness of the cellular transcription profiles at each time point during reprogramming, we performed PCA, and hierarchical clustering from 3615 genes. By comparing extracted genes to all genes contained in the microarray probe, the reprogramming trajectory can be traced from the extracted genes through PCA (Fig. 2a, Additional file 3: Figure S1a). Gene filtering system successfully increased the contribution ratio of PC1 and PC2 from 26.07% and 11.87% to 40.53% and 17.48%, respectively (Fig. 2a, Additional file 3: Figure S1a), supporting the technical validation of gene extraction filtering methods.

According to the PCA and HCA results, the transcriptome during cellular reprogramming was broadly divided into three clusters based on their similarities: the early phase from day 0 to 3, the mid phase from day 7 to 15, and the late phase from day 20 to later (Fig. 2). Although HA d15 was clustered within the late phase, this is consistent with a previous report that human astrocytes can be induced into iPSCs with high-efficient manner [38].

Notably, the results indicated that all reprogramming cell types exhibited uniformly greater dissimilarities in between the mid to late phase than in between the early to mid phase (Fig. 2).

### Unique gene expression patterns and functional annotations are conserved across different cell types during reprogramming

Next, to gain the functional insights of the gene expression dynamics during reprogramming, we clustered the dynamic patterns of gene expression into five groups and performed the functional annotations of gene enrichment and protein-protein interaction. The gene symbols in each cluster are listed in Additional file 4: Table S2.

The first cluster containing 816 genes had a higher expression in the early phase and remained suppressed throughout reprogramming process (Fig. 3a). These genes were mainly annotated as extracellular matrix organization, which could directly influence cell proliferation and differentiation [39]. Especially, the cluster included TGF-beta family members (TGFB1, TGFB1I1, TGFB2, TGFB3, TGFBI, TGFBR2, TGFBR3), and TGF-beta induced EMT markers (ZEB1, SNAI2, and TWIST2) (Additional file 4: Table S2). Evidently, these genes were reported as negative regulators of MET and downregulated by exogenous Sox2, Oct4, and c-Myc induction in MEFs reprogramming [40, 41]. Thus, these results suggest that the reprogramming cells from day 0 to 3 would prepare for MET, a prerequisite for reprogramming commencement, by inhibiting EMT pathways, which is one of the hallmarks of the initiation [3–5].

The second cluster genes had stable expression during the early and the mid phase but decreased patterns in the late phase (Fig. 3b). This cluster was annotated as immune response related genes, which can be caused by the effect of retroviral induction system for exogenous OSKM expression. Because OSKM transgenes were sustainably expressed by day 15 [29], and retroviral gene induction system is known to trigger innate immune response [42], OSKM retrovirus might attribute to upregulate immune system from early to mid phase of reprogramming. Notably, the suppression of the immune response by supplementation of either B18R interferon inhibitor or NFkB inhibition enhanced hiPSCs generation [43, 44], indicating the inverse correlation between immune system and reprogramming efficiency. Therefore, considering that interferon induced IFIT protein family was enriched in the early phase from the first gene cluster analysis (Fig. 3a), the innate immune related gene sets in the first and second clusters may have an inhibitory role of cellular reprogramming especially in the case of retrovirus induction system.

The gene expressions in the third cluster were transiently upregulated only in the mid phase, which were enriched by hemidesmosome and epidermal development related genes (Fig. 3c). This cluster included SFN and KRT6A, consistent with the previous report that epidermis related genes had a transient upregulation during the reprogramming of MEFs [6]. Given that the inhibition of these genes precedes the following activation of pluripotency genes at the late phase [6], the transitory expression of epidermis related genes could be implied as an important feature of the mid phase.

The genes in the fourth cluster had a sharp upregulation in the late phase of reprogramming, which were annotated as trans-synaptic signaling related genes (Fig. 3d). Interestingly, previous studies reported that neuronal stem cells (NSCs) can be reprogrammed by OCT4 single gene induction in both human and mouse because NSCs endogenously express Sox2, Klf4, and c-Myc [18, 45], indicating higher reprogramming efficiency of trans-synaptic enriched cell types. Considering that tissue-derived human neuronal progenitor cells were more closely related to ESCs/iPSCs compared with other tissue-derived cells (Additional file 5: Figure S2), it can be speculated that NSCs would share similar gene profiles to the late phase of human reprogramming cells.

The genes in the fifth cluster were gradually increased as the reprogramming progressed (Fig. 3e). They were highly annotated as cell cycle related genes, with especially dense protein-protein interactions and contained families of Cyclin (CCNA2, CCNB1, CCNB2, CCND2, CCNE1, CCNI2) and CDK (CDK1, CDK18, CDKN3) (Additional file 4: Table S2). This is in agreement with the previous study that hESCs/hiPSCs require high proliferation rate for the acquisition and maintenance of pluripotency and self-renewal [46]. The results may propose a possibility of positive selection during reprogramming, that is, a certain cell population which acquires high proliferating ability can survive in the early or/and mid phase, and thus would eventually become dominant in the late phase.

Because gene expression regulation is often linked with epigenetic alteration, we also investigated how the expression patterns of 3615 genes are coupled with epigenetic changes. To this end, we referred one published data of histone modification change during reprogramming in human fibroblasts by analyzing H3K79me2 and H3K27me3 as active and repressive marks of transcription, respectively [37]. We firstly examined 3 genes, SNAI2, TUBB3, and PRDM14, from different clusters with different expression patterns (Fig. 3, Additional file 2: Figure S3a), and as expected the changes of H3K79me2 and H3K27me3 in these loci during reprogramming tend to correlate with the expression patterns (Additional file 2: Figure S3a). Next, to determine the general modification patterns of H3K79me2 and H3K27me3 in each clustered gene during reprogramming, we counted the temporal changes of gene numbers with the histone marks in all 5 clusters (Additional file 2: Figure S3b). The number of active marked genes increases in cluster 1 and 2 but decreases in cluster 4 and 5, whereas the number of repressive marked genes increases in cluster 1 and 2 but decreases in cluster 4 and 5. Overall, these results suggest that our clustering based on gene expression change across the five types of cells can also reflect epigenetic changes, regardless of the different starting cells types and time points of the analysis.

### TF influence drastically changes in between the mid phase and the late phase

**Fig. 4:**
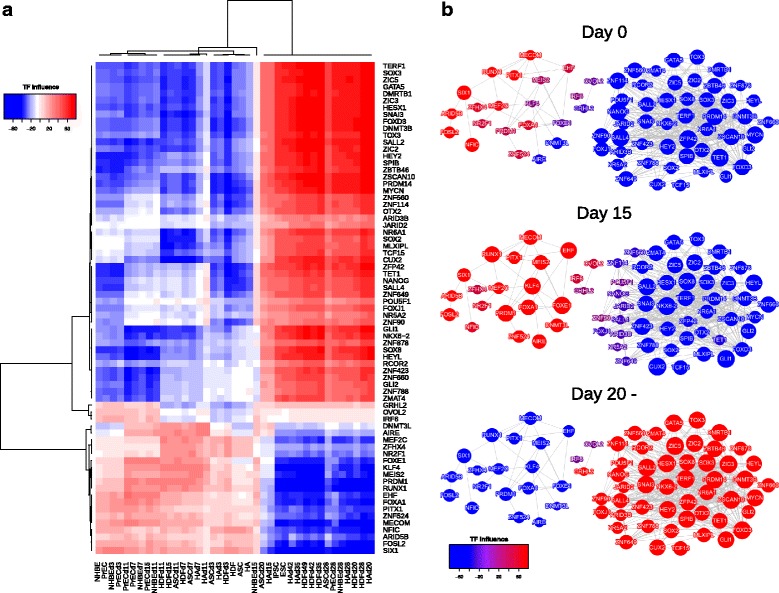
Dynamic activity of TF networks. The TF influence scores were indicated by the color (red:positive, blue:negative). **a** Heatmap of TF influences. **b** Co-regulatory TF networks during the reprogramming process

Since the gene expression patterns were primarily regulated by TFs, we scored influences of TFs and reconstructed TF network. We extracted 71 TFs with major influence and displayed the influences by colors. The heatmap of TF influences clearly exhibited two distinct clusters. The pluripotency-related TFs such as NANOG, SALL4, endogenous POU5F1 and endogenous SOX2 were the positive influence in the late phase. On the other hand, tissue morphogenesis associated TFs such as EHF, MEF2C and FOXE1 had the positive influences in the early phase (Fig. 4a). Next, we visualized the co-regulatory network of the 71 TFs for each time point of the reprogramming process. The time-course TF network illustrated that the positive influence TFs from day 0 and 15 had a sparse network compared to the negative influence TFs, whereas positive influence TFs from day 20 network was denser than negative influence TFs. This would reflect the heterogeneous cell status in different phases (Fig. 4b). Furthermore, no co-regulatory networks were observed between positive influence TFs and negative influence TFs in all phases, and particularly between the mid phase and the late phase (Fig. 4b). Therefore, these results suggested that the transition of TF influence occurred in between the mid phase and the late phase.

## Discussion

### Maturation could be the major roadblock of reprogramming in various human somatic cell types

In this study, we analyzed 3615 extracted genes with dynamic expression during reprogramming process from five human cell types (Fig. 1) and addressed that shared reprogramming route exists in human cellular reprogramming. The transcriptome analysis of cellular states similarity indicated that a common route of reprogramming process in human somatic cells was divided into early, mid and late phase with the major dissimilarity in between the mid and the late phase (Fig. 2). Moreover, we functionally annotated the groups of genes and clustered them by their gene expression patterns (Fig. 3). Finally, we reconstructed TF networks and revealed that the major difference of TFs activity occurred in transition between from the mid phase to late phase (Fig. 4). Overall, these results indicated that the maturation could be the major roadblock in reprogramming for not only human dermal fibroblasts [30] but also for various human cell types (Figs. 2 and 4).

In HDFs, maturation stage was reported to obstruct reprogramming procedure, which in turn could reduce the overall reprogramming efficiency [30]. For example, although about 20% of retroviral infected cells at day 7 of OSKM induction, expresses TRA-1-60, one of the pluripotent stem cell surface markers, only a small portion of the TRA-1-60 positive cells become iPSCs, because many intermediate cells revert back to TRA-1-60 negative cells [30]. However, our data clearly suggest that the major roadblock of reprogramming does not specifically depend on the cell type, but depends on the stage of the reprogramming, i.e. the maturation phase. This means then that the reprogramming is directed by stage specific manner rather than cell type specific manner. Evidently, our data well illustrated the common route of reprogramming from 5 different cell types. Of note, these 5 starting cells were derived from all different germ layers (HDF and ASC from mesoderm, HA from ectoderm, NHBE and PrEC from endoderm), suggesting that reprogramming process is not simply reversing the cell origin. Therefore, this highlights our finding that unique reprogramming pathway is shared in many different cell types; unlike normal development pathways are not conserved amongst different germ layers.

Notably, the transcriptome and TF activity of epithelial cells exhibited the distinct differences between the mid phase and the late phase, corresponding to maturation and stabilization (Figs. 2 and 4) even though epithelial cells do not require MET in the initiation. Therefore, studying of underlying mechanisms of maturation in more detail is important considering various human tissues derived cells become available in the clinical situation.

### Comparison of the results with previous research

Maturation was firstly described as the phase when the pluripotency genes such as endogenous Pou5f1, Nanog, and Sall4 begin to express [3, 5]. Because epigenetic modification is largely reported to play pivotal roles in the expression of pluripotency genes, reprogramming suppressors or enhancers through epigenetic changes were often stated in fibroblasts of mouse or human [30, 37, 47–52]. Interestingly, mouse B cells and mouse neural stem cells were also reported to have the obstructive effects of reprogramming during maturation stage, which was overcame by reprogramming enhancers [53–55]. For example, C/EBP-alpha overexpression in mouse B cell induces the expression of the dioxygenase Tet2 and promotes Tet2 binding to regulatory regions of pluripotency genes, which in turn highly accelerates reprogramming efficiency [53]. In addition, Tet1, Tet2 and Mbd3 work as facilitators of the reprogramming in mouse neural stem cells through upregulating pluripotency genes [54, 55]. However, these studies approached the issue of maturation stage by dealing with only small number of genes (C/EBP-alpha, Tet1, Tet2 and Mbd3), whereas our study addressed the importance of maturation in a larger scale using transcriptome analysis from five different human cell types. Therefore, our study is the first report to suggest that maturation can be a common roadblock of reprogramming process among human cell types derived from different germ layers.

Furthermore, our study could provide some candidate functional genes related to maturation, as the downregulation of high positive influence TFs in the early phase to the mid phase might have a key to overcome the roadblock to the maturation. For example, a recent study reported that co-expression of FOSL2 with OSKM had an inhibitory effect on the reprogramming of both of human corneal epithelial cells (CECs) and HDFs [56]. Similarly, our study showed the expression and influence of FOSL2 remained upregulated in the early and mid phase in both mesenchymal cells and epithelial cells but negatively regulated in the late phase (Fig. 4, Additional file 6: Figure S4), supporting that the inhibition of Fosl2 expression might drive reprogramming towards maturation phase. Interestingly, AP-1 complexes, c-Jun and Fos were reported to reduce the reprogramming efficiency in MEFs by impeding MET at initiation [57], yet, our results suggested that FOSL2 might have a suppressive role in maturation of reprogramming too.

In addition, DNMT3L, a catalytically inactive regulatory factor of DNA methyltransferases, was reported that it was highly expressed on day 20 of reprogrammed HDF in iPSCs generation [58]. Moreover, DNMT3L-overexpressing HeLa cells exhibited iPSC-like colonies and high SOX2 expression level, after over 20 passages [59]. However, the functional role of DNMT3L has not been studied yet in the context of cellular reprogramming to the best of our knowledge. Surprisingly, in our study, DNMT3L expression was transiently upregulated in the mid phase (Fig. 4, Additional file 7: Figure S5), indicating DNMT3L may act some biological role to facilitate maturation during reprogramming. Moreover, AIRE, exerted its expression and influence in the similar manner to DNMT3L, only positive in the mid phase (Fig. 4, Additional file 7: Figure S5). Given that the genomic locations of DNMT3L and AIRE are closely coordinated on chromosome 21 in human and they share their 23.5 kb upstream region, it can be speculated that DNMT3L and AIRE may be regulated by the same mechanisms such as transcriptional regulation or epigenetic modification. Especially, the dynamical changes of epigenetic states during reprogramming could be related to the suppression of cell-type-specific genes and activation of pluripotency genes. A recent study indicated that Polycomb Repressive Complex 2 (PRC2) is involved in the repression of fibroblast-specific genes through adding H3K27me3 in mouse and human fibroblasts, and yet involved in the activation of pluripotency genes [47]. Because DNMT3L can directly interact with PRC2 in mESCs [60], it could be speculated that DNMT3L supports epigenetic state via PRC2 during reprogramming process. Future studies to understand the biological roles of FOSL2 and DNMT3L will contribute to accelerate maturation and increase reprogramming efficiency in hiPSCs generation.

### Comparison of the reprogramming process between mouse and human

The previous studies illustrated the mouse cell line reprogramming from MEFs; firstly mesenchymal gene expression was lost, followed by transiently upregulation of epidermal genes, and lastly pluripotency genes are stably expressed [6, 7]. Interestingly, our study of human cellular reprogramming analysis were partially consistent with the mouse reprogramming gene expression patterns (Fig. 3a–e). Particularly, the TFs network suggested that epidermis related TFs such as KLF4 and EHF had a cooperative network, whose influence changed from positive to negative at the late phase (Fig. 4b). Several studies reported the significance of Klf4 in reprogramming efficiency; low protein level of Klf4 paused reprogramming process in MEFs regardless of high expression of other reprogramming factors, Oct4, Sox2 and c-Myc [61]; and the length of Klf4 isoforms was critical to determine efficiency of reprogramming [62, 63]. Therefore, KLF4 and its co-operative genes may play an important role in the intermediate process to direct to the late phase by overcoming the roadblock of reprogramming maturation. Furthermore, the transient upregulation of the epidermis related genes in human cells would support the possibility that reprogramming process could not be considered as a reversed process of the normal development [6].

### A possible population selection in maturation

Although, transcriptome dynamics during reprogramming were justifiably represented by using microarray dataset, because the microarray is bulk measurements on cell populations so it can mask the transcriptomic changes of small cell population [64]. Nevertheless, this study consistently revealed that the expression of cell cycle related genes gradually increased from the early phase to the late phase (Fig. 3e) and the TF influence was drastically changed between the mid phase to the late phase (Fig. 4a). In addition, the high density of TF network showing influence shift from negative to positive suggested the homogenous co-operative TF activity (Fig. 4b), strengthening the possibility that masked population could represent cellular reprogramming. Given that the reprogramming cells acquire the high proliferation ability at the early phase [46], only the small subset of cells which acquired pluripotency and high proliferation ability in the mid phase could survive and continue to proliferate in self-replication manner, which eventually dominated the late phase population. To address this issue accurately, single-cell RNA sequence at the mid phase would be required [64].

In comparison to microarray, RNA-seq would be more suitable to detect dynamically expressed genes because it is more sensitive in detecting genes with very low expression and has a wider dynamic range [65]. However, to our best knowledge, previously conducted time-course RNA-seq during reprogramming in human cells was focused on fibroblasts as the starting cells [66, 67], and not any other cell types. Therefore, the analysis of RNA-seq data is the subject of future study, when other cell type RNA-seq data to examine reprogramming procedure are reported.

It is also possible that the different copies of OSKM retroviral vectors were integrated into genome and influence different gene expression profiles. Indeed, previous study showed each iPS clone derived from MEF has different numbers of retroviral integration [68]. In addition, a subset of OSKM-induced MEFs become similar to extraembryonic endoderm stem cells (iXENCs) and the iXENCs tend to have lower viral insertions than iPSCs [69]. Considering that the copy numbers of OSKM retrovirus are different even within the same cell type (MEF) and that it may affect reprogramming states, the differences of virus integration among different cell types could be higher. However, regardless of different copy numbers of OSKM, our results consistently indicated that parts of reprogramming process could be shared among different cell types at least in human cellular reprogramming.

As far as we know, our report is the first study to describe that human reprogramming process was partially shared across multiple different human somatic cells and that maturation could be the common barrier in reprogramming in various human cell types. The strategy can be applied not only transcriptome but also epigenetic or proteomic studies and it would provide further insights of the fundamental mechanisms of cellular reprogramming.

## Conclusions

In summary, we illustrate that the reprogramming process was shared in five human somatic cell types by applying the genome-wide analyses of time-course microarray data. From the results of functional annotations of the gene expression patterns and reconstruction of transcription factor activity, we suggest the maturation could be the common roadblock of reprogramming into hiPSCs in various cell types. Identification of a reprogramming route shared in cell types would provide the keys to further investigate and understand the mechanisms of cellular reprogramming.

## Additional files

Additional file 1: Table S1. List of microarray sample data which used in this study. (TSV 5 kb)

Additional file 2: Figure S3. Epigenetic modification during reprogramming. **(a)** Gene expression patterns during reprogramming in five cells and H3K79me2 and H3K27me3 ChIP-seq tracks (red and blue, respectively) for SNAI2, TUBB3, and PRDM14 in fibroblasts (D0), at day 6 of OSKM induced fibroblasts (D6) and ESCs (ES). Bars below each ChIP-seq track were genomic features which contain top 0.1% signal intensity. **(b)** Gene expression patterns in each cluster (the same as Fig. 3) and the number of genes with the histone marks among genes in each cluster. (PDF 140 kb)

Additional file 3: Figure S1. PCA and HCA of each cell type by using log_2_ expression value of all 22,062 genes in GPL14550 platform. (PDF 70 kb)

Additional file 4: Table S2 List of gene symbols clustered with five groups in Fig. 3. (TSV 24 kb)

Additional file 5: Figure S2. PCA of 75 cell types by using log_2_ expression value. **(a)** all 22,062 genes in GPL14550 platform. **(b)** extracted 3615 genes. Tissue-derived cells and ESC-derived cells were labeled as black and dark red, respectively. (PDF 66 kb)

Additional file 6: Figure S4. FOSL2 gene expression pattern. (PDF 39 kb)

Additional file 7: Figure S5. DNMT3L and AIRE gene expression patterns. (PDF 76 kb)

## Abbreviations

ASC: Adipose-derived stem cell
EMT: Epithelial-mesenchymal transition
HA: Human astrocyte
HCA: Hierarchical cluster analysis
HDF: Human dermal fibroblast
iPC: Induced pluripotent stem cell
MEF: Mouse embryonic fibroblast
MET: Mesenchymal-epithelial transition
NHBE: Normal human bronchial epithelial cell
NPC: Neural progenitor cell
NSC: Neural stem cell
PCA: Principal component analysis
PrEC: Prostate epithelial cell
TF: Transcription factor
