# Supplementary figures and images for: Time-course transcriptome analysis of human cellular reprogramming from multiple cell types reveals the drastic change occurs between the mid phase and the late phase
Source: BMC Genomics. 2018 Jan 3;19:9. doi: 10.1186/s12864-017-4389-8 (PMC5753469; doi:10.1186/s12864-017-4389-8)

**a**

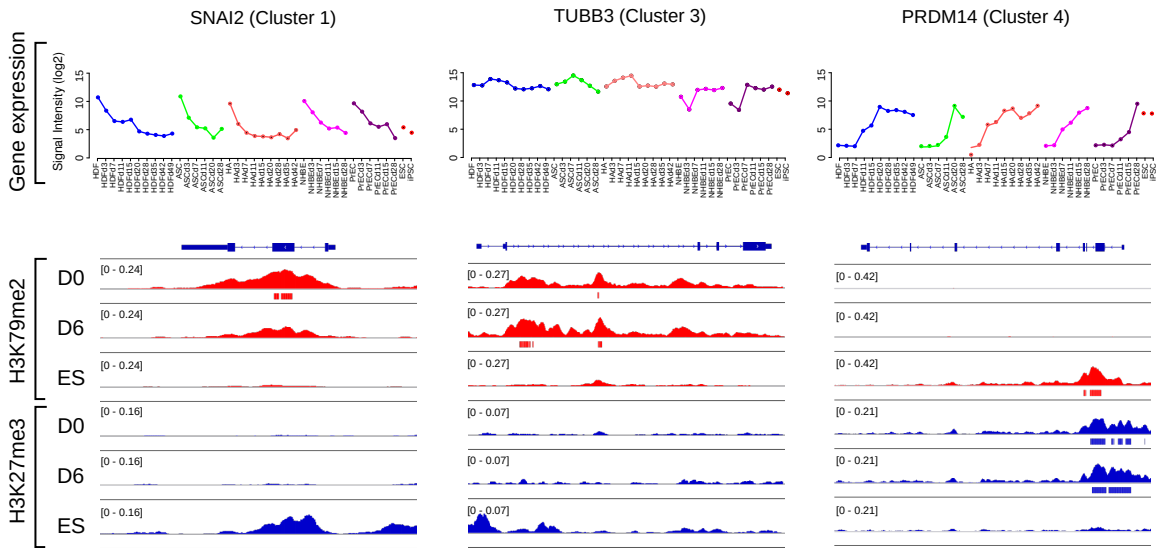**b**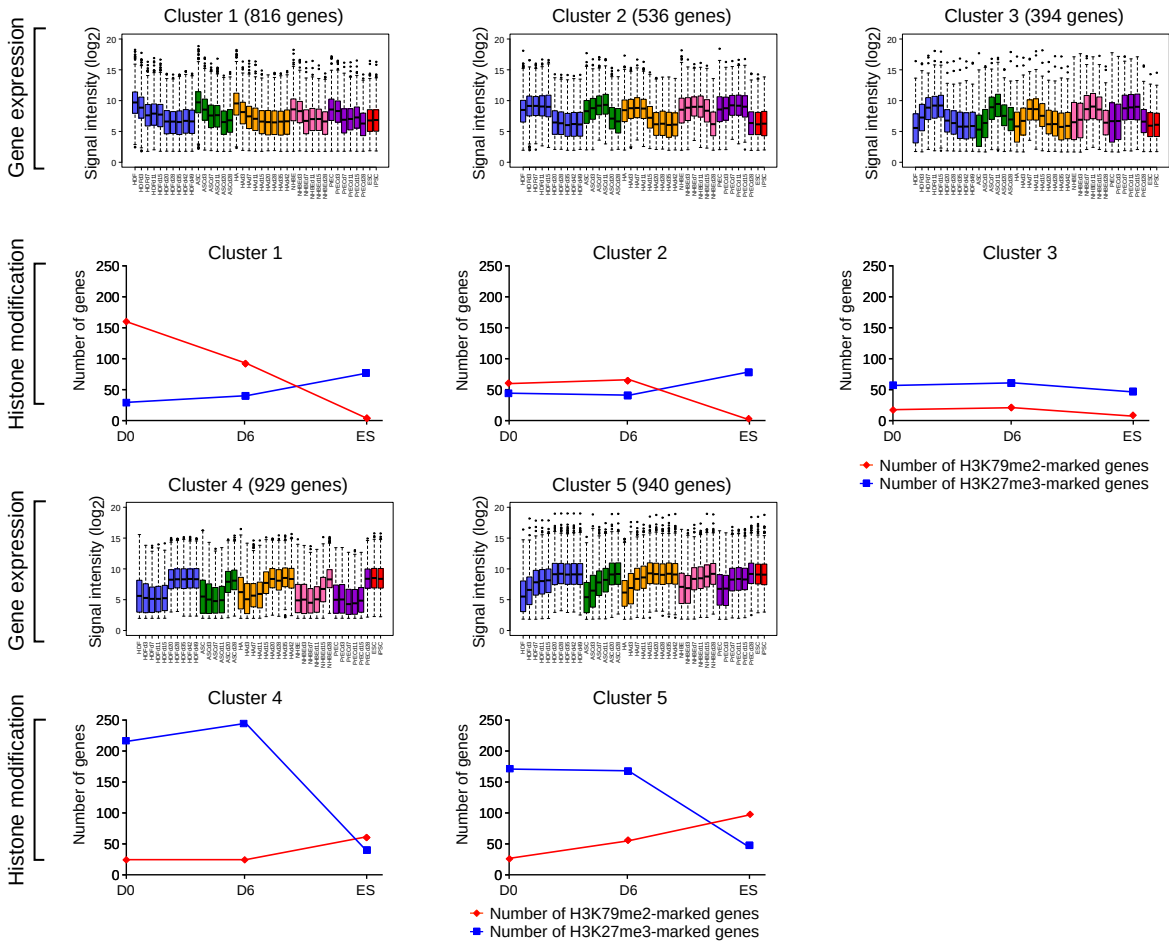

Supplement: Supplementary file 2 — Epigenetic modification during reprogramming. (a) Gene expression patterns during reprogramming in five cells and H3K79me2 and H3K27me3 ChIP-seq tracks (red and blue, respectively) for SNAI2, TUBB3, and PRDM14 in fibroblasts (D0), at day 6 of OSKM induced fibroblasts (D6) and ESCs (ES). Bars below each ChIP-seq track were genomic features which contain top 0.1% signal intensity. (b) Gene expression patterns in each cluster (the same as Fig. 3) and the number of genes with the histone marks among genes in each cluster. (PDF 140 kb) [file 12864_2017_4389_MOESM2_ESM.pdf]

**a**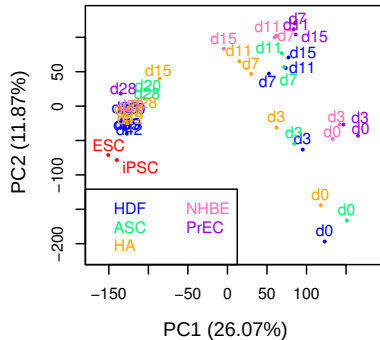**b**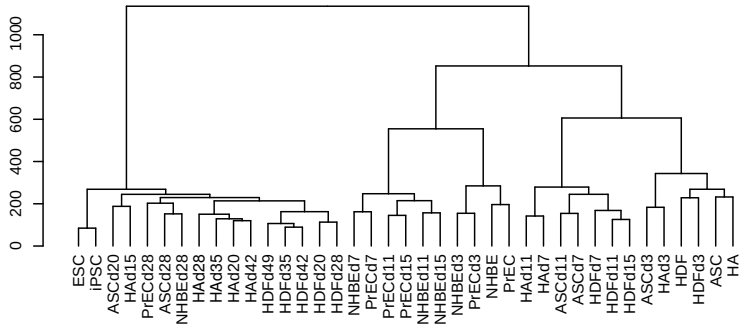

Supplement: Supplementary file 3 — PCA and HCA of each cell type by using log2 expression value of all 22,062 genes in GPL14550 platform. (PDF 70 kb) [file 12864_2017_4389_MOESM3_ESM.pdf]

**a**

### All 22062 genes

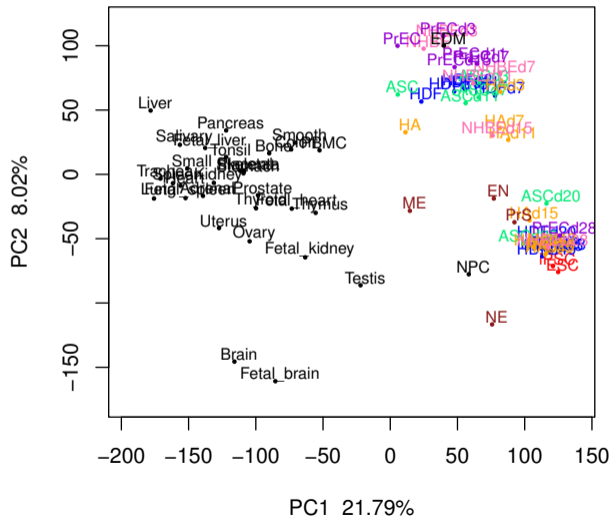**b**

## Extracted 3615 genes

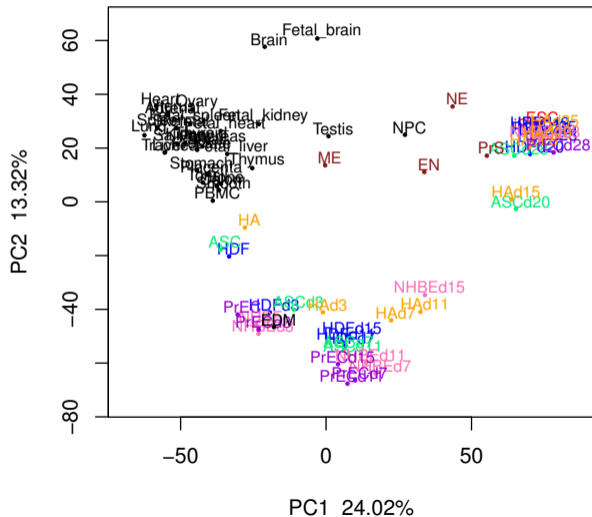

Supplement: Supplementary file 5 — PCA of 75 cell types by using log2 expression value. (a) all 22,062 genes in GPL14550 platform. (b) extracted 3615 genes. Tissue-derived cells and ESC-derived cells were labeled as black and dark red, respectively. (PDF 66 kb) [file 12864_2017_4389_MOESM5_ESM.pdf]

# FOSL2

A\_23\_P348121

Signal Intensity (log2)

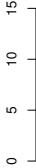

Supplement: Supplementary file 6 — FOSL2 gene expression pattern. (PDF 39 kb) [file 12864_2017_4389_MOESM6_ESM.pdf]

Signal Intensity (log2)

## DNMT3L

A\_23\_P17673

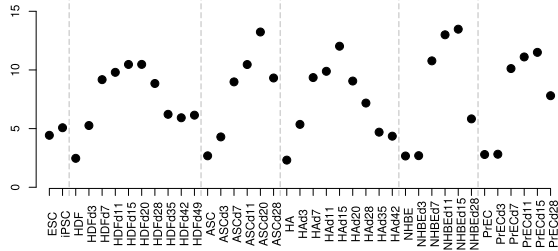

Signal Intensity (log2)

## AIRE

A\_23\_P68740

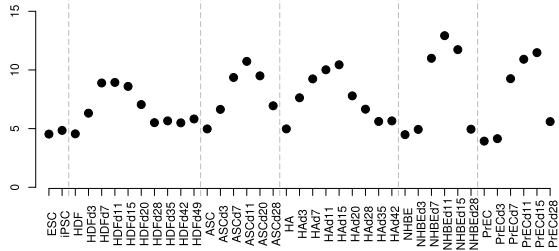

Supplement: Supplementary file 7 — DNMT3L and AIRE gene expression patterns. (PDF 76 kb) [file 12864_2017_4389_MOESM7_ESM.pdf]
